# Simulation research on evacuation of public buildings based on BIM technology and fuzzy algorithm

**DOI:** 10.1038/s41598-026-53101-6

**Published:** 2026-05-15

**Authors:** Chenpu Zhang, Fengtao Liu

**Affiliations:** 1 Minxi Vocational and Technical College, Longyan City, Fujian China; 2 Liuzhou Institute of Technology, Liuzhou, Guangxi China

**Keywords:** Fire evacuation, Fuzzy algorithm, BIM technology, Factors of evacuating personnel, Evacuation simulation, Engineering, Environmental social sciences, Mathematics and computing

## Abstract

Large public buildings are characterized by high occupancy and complex functions, which can easily lead to issues such as congestion in evacuation routes and disorderly crowd behavior in the event of a fire. Conducting analyses and simulation assessments of evacuation mechanisms in fire scenarios is of great significance for improving building fire safety standards and emergency management capabilities. At present, there are significant gaps in the existing research on evacuation simulation for public buildings: Most studies rely on fixed parameter assumptions and fail to effectively quantify the influence of subjective factors such as psychological factors, safety awareness, and social roles on evacuation behavior. Moreover, the combination of BIM technology and evacuation simulation mostly focuses on the presentation of spatial geometric information, lacking a deep integration with quantitative methods for quantifying the subjective behavior of personnel, resulting in insufficient authenticity and predictive reliability of evacuation simulations, and making it difficult to precisely support fire protection design and emergency decision-making. In response to this research gap, this study has established an integrated framework that quantifies subjective human-related factors, maps them to key behavioral parameters through fuzzy inference, and couples them with BIM-based fire and evacuation simulations to provide a verifiable linkage between fire scene constraints, human behavior, and evacuation outcomes. This paper employs fuzzy logic theory together with Pyrosim and Pathfinder to investigate the effects of human-related subjective factors and fire scene conditions on fire evacuation safety. A questionnaire survey was conducted to examine how psychological factors, safety awareness, and social roles of pedestrians influence evacuation behavior. Through the reliability and validity test of the valid questionnaire data and the spearman correlation analysis, it is found that there is a significant positive correlation between safety awareness, psychological factors, social roles and the evacuation behavior. Based on fuzzy rules, the domains and membership functions of the linguistic variables representing these factors are defined, enabling the quantification of the influencing factors and the calculation of the initial evacuation speed. Finally, a BIM model was established and applied to the evacuation simulation of a large shopping mall project in Southwest China to verify the feasibility of the fuzzy algorithm and the safety of the evacuation design. This research innovatively combines fuzzy algorithms with BIM technology, making up for the deficiencies of existing studies in the quantification of subjective factors of personnel and the deep integration of BIM technology. It provides a more scientific calculation plan and data for the study of public building evacuation, and offers reference basis for fire protection design, personnel allocation, emergency plan formulation, and rescue operations.

## Introduction

With rapid economic development and urbanization, many large public buildings, such as shopping centres, railway stations, and sports stadiums, have emerged in China to meet the growing needs of urban life, transportation, and public services. These buildings tend to have increasingly complex layouts, higher levels of enclosure, and dense occupant loads, which in turn pose greater challenges to fire safety^1^. The Global Agenda for Sustainable Architecture and Urban Development issued by the United Nations suggests that emphasis should be placed on the enhancement of emergency response capabilities in urban construction. The Ministry of Housing and Urban-Rural Development (MOHURD) issued the Information Technology Development Plan for the Construction Industry (2022–2025) in 2022, which emphasizes the importance of the application of BIM technology in building design and management, especially in improving building safety and emergency management^2^.

In recent years, as public buildings transition from the construction phase to the operation and maintenance phase, the role of facility management in ensuring building safety, emergency response, and evacuation organization becomes increasingly prominent. Integrating evacuation simulation with building operation and maintenance management requirements helps provide more actionable technical support for risk governance and emergency decision-making in public buildings^3^. The rapid development of building information modeling (BIM) technology has provided new perspectives and tools for building design and management^3^.By digitally modeling buildings and integrating rich building information, BIM technology can not only accurately present the building structure and function, but also simulate the behavior of crowds in different situations and provide data support for the development of evacuation strategies^4^. In addition, the visualization characteristics of BIM enable building designers and managers to intuitively identify potential safety hazards, so as to optimize the design scheme and improve the safety of the building.

Crowd evacuation behavior is affected by a variety of factors, including crowd density, individual behavior, and environmental characteristics. Relying solely on classical evacuation models, which include rule-based and network-flow approaches as well as pedestrian-dynamics models such as the social-force model and cellular automata, may limit predictive accuracy in high-density situations, especially during rapidly evolving emergencies where individual responses and decision-making become highly heterogeneous^5^. Fuzzy control algorithms, as a technique for handling uncertainty and fuzziness in information processing, enable fuzzy controllers to convert the ambiguity of crowd behavior into actionable decisions by setting fuzzy rules and reasoning mechanisms^6,7^. The 3D building model established by BIM technology is combined with fuzzy control algorithms. BIM technology provides the spatial layout of the building, the width of the passageway, and the safety facilities, which can more realistically reflect the behavioral characteristics of the crowd in the evacuation process.In the study of Elsayed et al. through the integration of the simulation of the dynamic behavior of the crowd in the BIM model, it is possible to update the evacuation path in real time, and adapt to the changes of the environment and thus enhance the effectiveness of evacuation strategies^8,9^.

This paper aims to enhance the realism and predictive reliability of fire evacuation simulations in large public buildings. By incorporating subjective human factors into the evacuation modeling process, it seeks to provide more robust support for optimizing evacuation designs and conducting safety assessments. First, identifying and quantifying subjective factors such as psychological factors, safety awareness, and individual role characteristics, and analyzing the mechanisms through which they influence evacuation behavior and decision-making processes. Second, constructing an evacuation simulation framework capable of mapping these subjective factors to key behavioral parameters, in order to evaluate evacuation efficiency and identify potential congestion points. Third, the validity of the simulation model is verified and optimization suggestions are made. Through systematic analysis and discussion of the simulation results, this paper evaluates the evacuation efficiency and safety of the constructed model in different scenarios and highlights the advantages of the integrated approach based on BIM and fuzzy control algorithms.

The structure of the paper is as follows: Sect.  2 reviews the current state of research both domestically and internationally; Sect.  3 constructs an evacuation simulation model based on BIM technology and fuzzy control algorithms, including data collection, setting control rules, and fuzzy inference mechanisms; Sect.  4 presents the experimental design and result analysis, setting up experiments through scenario simulations and data processing, and comparing the results of evacuation time and efficiency; Sect.  5 discusses the limitations of the model. Finally, the conclusion summarizes the findings and proposes directions for future research.

## Literature review

### The application of BIM technology in fire evacuation simulations

First, BIM-driven evacuation scenario construction and parameter extraction. Existing research typically follows a technical approach that includes BIM modeling, data export, and evacuation simulation platforms^10,11^. Elements such as door and window locations, corridor widths, staircase connections, and the number of exits are mapped from the BIM model to the simulation environment to enhance the geometric accuracy and reproducibility of evacuation modeling^12,13^. The contributions of such research lie in the followings. On the one hand, BIM reduces the manual modeling costs associated with constructing evacuation models^14^. On the other hand, it enhances the credibility of simulation scenarios through information consistency and supports rapid model updates in response to design changes^15^.

Second, data interoperability and coupled applications between BIM and fire dynamics simulation. In the field of fire simulation, research typically involves integrating BIM models with platforms such as FDS^16^. By utilizing information provided by BIM, such as spatial boundaries, partition walls, and functional zones which the modeling of fire source locations and detector placements is supported, enabling the correlation of input and output for key environmental parameters such as smoke dispersion, visibility, and CO concentration^17,18^. Mao et al. explicitly incorporated fire environment constraints into evacuation assessments, ensuring that evacuation safety determinations no longer rely solely on static capacity, and enabling the formation of a consistent analytical framework with time-based safety thresholds such as ASET and RSET^19,20^.

Third, data interoperability is the foundation for BIM-supported multi-software integration, and BIM-based evacuation simulation is rapidly converging with evacuation modeling, intelligent systems, and behavioral modeling. However, insufficient cross-platform interoperability, data loss during transmission, and the difficulty of effectively incorporating simulation results remain key bottlenecks limiting its engineering applications^21^. To address this issue, existing research has achieved bidirectional data transfer between Revit and Pathfinder by expanding the IFC property set and developing dedicated plugins, thereby demonstrating the feasibility of BIM in a closed-loop process encompassing model input, simulation calculations, and result feedback^22^. Building on the foundation of data interoperability, research has shifted toward semantic collaboration and multi-party information sharing. Focusing on information exchange and coordinated response, research has advanced standardized information sharing for users, facility managers, and fire and rescue personnel by expanding the IFC standard and implementing automated parameter processing mechanisms^23^. The development of a collaborative fire emergency response system that integrates BIM geometric data with indoor positioning technology demonstrates that a unified digital environment can enhance the efficiency of route planning, information sharing, and emergency coordination, while reducing computation time and positioning errors^24^. The deep integration of BIM and IoT data has further advanced research on dynamic evacuation. By incorporating a fire evacuation ontology and a dynamic knowledge graph, related studies have provided a semantic data foundation for dynamic path planning, demonstrating that BIM can not only store static building information but also support evacuation decision-making information that is continuously updated as the fire situation evolves^25^. By importing BIM models into Pyrosim and Pathfinder in formats such as CAD, we have achieved joint calculations for fire environment simulation and evacuation analysis, demonstrating that BIM can effectively support rapid data exchange and coupled analysis across multiple software platforms^26^. The value of BIM in engineering extends beyond improved modeling efficiency; it also lies in its ability to ensure that simulation scenarios are rapidly updated in real time as designs change, parameters are adjusted and fire conditions evolve^27^.

### Application of fuzzy algorithm in evacuation simulation

Fuzzy control algorithm is a control method based on fuzzy logic, which can effectively simulate the decision-making process of complex systems. In evacuation simulation, fuzzy control algorithms are being able to model and optimize the behavior of crowds^28,29^. The fundamental principle of fuzzy control is grounded in human decision-making processes, using fuzzy sets and fuzzy rules to represent system knowledge. The concept of fuzzy sets was first introduced by Zadeh, laying the foundation for the development of fuzzy logic^30^. Belman-Flores explored the problem of stability of fuzzy control systems, and proposed a control design method based on fuzzy logic. These studies provided theoretical support for the application of fuzzy control algorithms^31^.

In evacuation simulation research, fuzzy control algorithms are effective in capturing the dynamic behavior of crowds and simulating behavioral characteristics under different evacuation conditions^32,33^. Fuzzy control can optimize the evacuation path and improve the evacuation efficiency^34^. Sathiya combined fuzzy logic with reinforcement learning to develop a novel evacuation model, which adjusts evacuation strategies in real time using live data to respond to emergencies^35^. Soltaninejad applied fuzzy control algorithms in fire evacuation scenarios and constructed a decision support system that comprehensively considered the density of the crowd, the escape time and the exit availability of a decision support system, which concluded that fuzzy control can significantly improve evacuation efficiency and reduce crowd congestion^36^. Meanwhile, Nazif studied the adaptability of fuzzy control under different crowd characteristics and proposed a personalized evacuation strategy based on fuzzy control to meet the needs of different individuals^37^.

Fuzzy algorithms are employed to quantify subjective human factors, necessitating a technical pathway encompassing measurement, quantification, inference, verification. Accordingly, this paper utilizes questionnaires to measure subjective factors, enhancing the rationality of variable quantification and model credibility through reliability and validity testing alongside simulation results.

### Evacuation modeling for public buildings

Evacuation in public buildings presents greater complexity, primarily due to high occupancy densities and significant fluctuations in occupant distribution, as well as the fact that most occupants are unfamiliar with the building environment. Consequently, evacuation in public buildings is not merely a matter of spatial circulation, but rather a complex systemic issue resulting from the interplay of the built environment, crowd behavior, and emergency response coordination. Based on this, research on evacuation models for public buildings has gradually shifted toward a more diversified and integrated approach, modeling and analyzing evacuation processes from the perspectives of the built environment and evacuation performance assessment^38,39^. Lattice gas modeling provides data support for evacuation design. Ionescu used a lattice gas model to simulate the escape time of students in an emergency situation and to analyze the process of evacuation of students from a classroom. The model is able to recognize room usage patterns and expected escape times, thus giving the safest location in the room based on individual evacuation times^40^. Research by Tamang and colleagues further demonstrated that lattice gas models can accurately predict escape times across various evacuation scenarios^39,40^. In addition, Stock found that effectively integrating real-time data with lattice gas models can significantly improve the accuracy and efficiency of evacuation route optimization^41^.

The Agent model is capable of capturing heterogeneous characteristics using individuals as the basic unit, making it highly applicable to research on evacuation in public buildings; its research focuses primarily on three aspects: individual differences, social interactions, and emotional responses^42,43^. On the one hand, relevant research has incorporated various demographic characteristics into agent-based modeling frameworks to simulate differentiated evacuation behaviors under emergency conditions. This enhances the models’ ability to capture complex population compositions and diverse response mechanisms^44^. On the other hand, as attention to the complexity of collective behavior has grown, factors related to social interaction have been introduced, indicating that interactions among individuals significantly influence route selection, following behavior, and the evolution of local congestion^45^. In addition, emotional factors have gradually been incorporated into agent-based models. Research findings indicate that changes in individual emotions not only influence evacuation decisions but also affect overall evacuation efficiency through group contagion effects, thereby providing a new analytical perspective for modeling behavior in complex scenarios^46^.

In studies of evacuation from public buildings, cellular automaton models focus on the evolutionary patterns of crowds driven by local rules. Regarding the formation of congestion, cellular automaton models combined with game theory rules can effectively describe collision effects and competitive behavior in exit areas; appropriately adjusting the placement of obstacles near exits helps reduce the likelihood of conflicts among people and alleviates exit blockages^47^. With regard to exit conflicts, relevant studies indicate that corridor layout, bottleneck locations, and crowd aggregation patterns significantly influence the spread of congestion and changes in efficiency during evacuation; based on these findings, corresponding spatial optimization schemes have been proposed^48^. From the perspective of passageway design optimization, analyses of traffic efficiency under different crowd density conditions also indicate that passageway width and layout are key spatial variables that determine evacuation speed^49^. Above all, research on cellular automaton models focuses on revealing the mechanisms of congestion and the patterns of spatial organization through simplified rules; however, its ability to capture individual psychological differences, decision-making heterogeneity, and complex behavioral mechanisms remains relatively limited.

The integration of VR with intelligent modeling has enabled evacuation studies to incorporate immersive simulations and interactive training. Relevant research has applied VR technology to evacuation simulations in typical public settings, such as subways and large-scale events, demonstrating that this approach can realistically replicate passenger evacuation processes during emergencies while enhancing the flexibility and adaptability of simulation systems^50,51^. Immersive training enhances participants’ risk awareness and emergency response capabilities, thereby improving their preparedness to act in real-life disaster scenarios^52^.Research on VR models has focused primarily on scenario reconstruction, interactive training, and behavioral feedback; however, its limitations lie in its emphasis on simulation and demonstration functions, making it difficult to directly establish a system of analytical indicators that is repeatable, quantifiable, and suitable for engineering evaluation.

### Literature review summary

Existing research lacks solutions to achieve more integrated and efficient solutions in building evacuation management, especially to provide more flexible emergency decision support during emergencies. First, the integration and application of BIM with other emerging technologies is insufficient, especially in the emergency management and evacuation process, how to effectively combine BIM with real-time data and decision support systems to improve evacuation efficiency and safety has not been fully explored. Second, the application of fuzzy control algorithms in evacuation simulation is mostly concentrated on the basic principles of the algorithm and simple application scenarios, with a lack of in-depth analysis of complex scenarios and the interactive effects of multiple factors.Third, the evacuation models of public buildings mostly focus on the application of a single model, and there is a lack of in-depth discussion on combining the advantages of different models to realize more accurate prediction and effective strategy formulation in complex evacuation environments.

Based on this, the contributions of this paper are as follows:

1. Quantifying subjective variables such as psychological factors, safety awareness, and individual role characteristics through questionnaire measurement, and enhancing measurement reliability via reliability and validity testing;
2. Constructing a fuzzy inference rule library to map subjective factors onto key behavioral parameters like initial evacuation velocity, thereby addressing limitations of fixed-parameter assumptions;
3. Establishing highly realistic public building scenarios using BIM, coupled with Pyrosim fire simulation and Pathfinder evacuation simulation, to achieve a verifiable evaluation process for fire scene constraints, behavioral parameters, and evacuation outcomes, which enhances the engineering applicability and interpretability of evacuation simulations.

## Methods

### Methodological framework

The technical route of this study is to establish a progressive and integrated system of “quantification of subjective behavioral characteristics - parameterization of algorithms - modeling of simulation”, through clear parameter transmission, logical mapping and model embedding methods, to achieve deep coupling of questionnaire survey data, fuzzy control algorithms and BIM + Pyrosim + Pathfinder fire evacuation simulation, forming a complete technical closed loop of “data input - algorithm transformation - model application - result verification”.

Firstly, the survey questionnaire data obtained in this chapter is the data source for the design of fuzzy algorithm parameters. By extracting statistical characteristics of each indicator, matching the input and output dimensions of the algorithm, defining the domain and membership function parameters, subjective indicators such as physical health status and panic degree are transformed into quantifiable parameters that can be calculated by the algorithm, and the reasoning weights are set according to the strength of the correlation between the indicators to ensure that the algorithm parameters are in line with the actual behavioral patterns.

Secondly, through the different characteristic initial evacuation speeds output by the fuzzy algorithm, the core parameters of the next stage of the simulation model are guided. By dividing the simulated population according to the algorithm dimensions, accurately assigning the initial speed, and simultaneously combining the dynamic attenuation rules of the fire scene environment, this parameter is embedded in the Pathfinder model, replacing the traditional fixed speed parameters, achieving “subjective behavior + objective environment” dynamic speed modeling, and completing the core improvement of the traditional simulation model.

Finally, by integrating the evacuation model with subjective behavior parameters and the Pyrosim fire model, a three-in-one simulation scenario is constructed based on the BIM model of the shopping mall. Through multi-dimensional comparison with the traditional fixed speed model to verify the effectiveness of the model improvement, and at the same time, using the simulation results to reverse verify the scientificity of the questionnaire indicators and algorithm parameters, a logically complete, path clear, and traceable verification research technical route loop is formed.

Thus, in the next chapter of this study, the experimental design and result analysis will be carried out.

### Analysis of factors and mechanisms influencing evacuation behavior

In order to carry out the study of individual subject factors affecting fire evacuation in the event of a fire in a large public building, a questionnaire was used to collect data related to the subject of escape affecting evacuation behavior under different dimensions.

Relevant studies^53–55^ have shown that during fire emergencies in large public buildings, the evacuation speed of crowds is not only influenced by objective factors such as spatial layout and fire protection facilities, but is also significantly affected by individuals’ evacuation decisions. Therefore, this paper combines the research and analysis of related literature, and sets up four dimensions of psychological factors, safety awareness, evacuation behavior, and social roles from the perspective of the main body of the evacuating individual to study its influence on evacuation behavior. The designed questionnaire measurement scale is shown in Table 1.

**Table 1:** Variable measurement scale.

Variable	No.	Measurement items	Score
Psychology	P1	I tend to follow others during evacuation	Measured using a 5-point Likert scale, ranging from “Strongly Disagree” to “Strongly Agree,” corresponding to values from 1 to 5.
	P2	I feel panic during a fire emergency (or degree of panic)	
	P3	I exhibit a fluke mentality when a fire occurs	
	P4	I am likely to take risky actions during a fire	
Safety awareness	S1	I actively participate in fire evacuation drills	
	S2	I make a point of familiarizing myself with mall evacuation routes in advance	
	S3	I know how to use fire safety equipment	
	S4	I follow evacuation signs and directions during an emergency	
Evacuation behavior	E1	I am capable of leading others in evacuation	
	E2	If separated from my family or friends, I will return to search for them	
	E3	I will assist those calling for help during evacuation	
	E4	I will return to retrieve valuable belongings if lost	
Social role	R1	I believe others will take care of themselves, so I don’t need to be responsible	
	R2	Parents will prioritize their own children during evacuation	
	R3	In group outings, the organizer will be responsible for directing the evacuation	
	R4	Males should prioritize helping females during evacuation	

Table 1 is a differentiated design based on the actual impact of the behavioral or psychological factors measured by the variable items on fire evacuation. The item design strictly aligns with the conceptual essence and connotation dimensions of each variable, and does not rely on a “single measure of good or bad evacuation”, but rather takes “whether the item statement matches the actual psychological / cognitive / behavioral characteristics of the respondents” as the core scoring basis. The core of the evacuation behavior dimension is to comprehensively measure the actual behavioral tendencies of the respondents during fire evacuation, rather than solely measuring “positive evacuation behavior”. The connotation attributes of different variables (adverse/positive/neutral combination), and the internal connotation dimensions differences within the same variable, determine that the score level of the items naturally leads to different interpretations of “evacuation situation”. The scoring design of all items is to serve the precise measurement of the actual level of the corresponding variables, thereby guiding the judgment of the actual impact of this score on the evacuation effect, so that when formulating evacuation plans and management measures, these factors can be comprehensively considered, and targeted measures can be taken to ensure the efficiency and safety of evacuation. The selection of factors in this variable table is based on references to the literature^56,57^.

#### Descriptive statistical

The official distribution of the questionnaire in this paper is 100, and all the questionnaires including network questionnaires are returned 92, and 7 invalid questionnaires are found through screening (answering time less than 60 s or failing to pass the questionnaire-specific lie detector item). Finally, 85 valid questionnaires were obtained, with a valid questionnaire rate of 92.39%.

This study was conducted in accordance with the Declaration of Helsinki and all relevant ethical guidelines and regulations for human‑related research. All protocols involving questionnaire surveys were reviewed and approved by the Ethics Committee of Minxi Vocational and Technical College and Ethics Committee of Liuzhou Institute of Technology. Informed consent was obtained from all individual participants included in the questionnaire survey; for participants under 18 years of age, informed consent was obtained from their parents or legal guardians prior to data collection.

The 100 valid samples selected for this study were determined based on the classic criteria of social science research, the compatibility of the research design and statistical methods, and can meet the statistical requirements for the exploratory analysis of this study. It can lead to statistically significant conclusions. The core scientific basis is as follows:

Compliance with the sample ratio standard for scale research items^58^: This study has 16 observed items. According to the basic standard of the relevant literature that the sample size should be ≥ 5 times the number of observed items, 100 samples have met this standard range.

Scientific sampling ensures sample representativeness: This study adopts stratified random sampling, stratified by 5 core characteristics such as age, gender, physical condition, and education level. The representativeness of the sample core depends on the sampling method rather than just the scale^59^. The representativeness of the 100 stratified samples has met the representativeness of a simple random sample of the same scale, and can effectively reflect the overall characteristics.

The basic characteristics of the questionnaires are shown in Table 2.

**Table 2:** Sample statistical characteristics.

Indicator	Option	Frequency (person)	Percentage(%)	Skewness	Kurtosis
Gender	Male	47	55.29%	0.109	1.338
	Female	38	44.71%		
Age	Under 18	4	4.71%	0.317	3.221
	18–30	21	24.71%		
	31–45	35	41.18%		
	46–60	25	29.41%		
	Over 61	4	4.71%		
Physical Condition	Unable to evacuate independently	2	2.35%	0.476	4.187
	Able to evacuate with assistance	13	15.29%		
	Able to evacuate independently	70	82.35%		
Education Level	Primary school or below	3	3.53%	0.534	3.902
	Junior high school	8	9.41%		
	High school/Technical school	8	9.41%		
	College (Undergraduate/Diploma)	62	72.94%		
	Graduate (Master’s/Doctorate)	4	4.71%		
Evacuation training frequency	Never participated	42	49.41%	0.335	4.098
	Two times or fewer	28	32.94%		
	More than two time	15	17.65%		

The absolute value of skewness of all measurement question items is less than 3, and the absolute value of kurtosis is less than 7, which is consistent with normal distribution. The five basic information items of gender, age, physical health status, education level, and number of fire evacuations attended passed the ANOVA test with the raw data in Table 3, implying that the different base samples showed consistency for all the question items, which meets the requirements for further data analysis.

**Table 3:** Descriptive statistical analysis of samples.

Indicator	No.	*N*	Minimum	Maximum	Mean	Standard Deviation	Skewness	Kurtosis
Psychology	P1	85	1	5	3.43	1.17	-0.56	-0.17
	P2	85	1	5	2.56	1.11	-0.33	-0.89
	P3	85	1	5	2.93	0.88	-0.62	-0.22
	P4	85	1	5	2.37	0.81	-0.33	-0.23
Safety awareness	S1	85	1	5	2.89	1.05	-0.62	-0.63
	S2	85	1	5	3.22	0.85	-0.29	-0.59
	S3	85	1	5	2.32	0.93	-0.61	-0.47
	S4	85	1	5	2.79	0.87	-0.48	-0.86
Evacuation behavior	E1	85	1	5	3.7	0.93	-0.39	-0.2
	E2	85	1	5	3.57	1.14	-0.66	-0.32
	E3	85	1	5	3.03	0.92	-0.33	-0.84
	E4	85	1	5	3.22	0.83	-0.66	-0.44
Social role	R1	85	1	5	3.95	1.15	-0.3	-0.48
	R2	85	1	5	2.94	0.8	-0.27	-0.7
	R3	85	1	5	3.76	1	-0.27	-0.23
	R4	85	1	5	3.77	0.82	-0.6	-0.3

**Table 4:** Reliability analysis of variables.

Indicator	No.	CITC	Cronbach’s Alpha if item deleted	Cronbach’s Alpha
Psychology	P1	0.72	0.771	0.733
	P2	0.751	0.855	
	P3	0.755	0.922	
	P4	0.726	0.882	
Safety awareness	S1	0.783	0.814	0.818
	S2	0.785	0.867	
	S3	0.799	0.855	
	S4	0.724	0.939	
Evacuation behavior	E1	0.804	0.776	0.763
	E2	0.724	0.863	
	E3	0.721	0.937	
	E4	0.741	0.773	
Social role	R1	0.815	0.804	0.751
	R2	0.715	0.926	
	R3	0.84	0.973	
	R4	0.755	0.787	

The descriptive statistics of the four variables in the relational model with 16 question items are shown in Table 4. The maximum absolute value of skewness for all samples is 0.66 < 3, and the maximum absolute value of kurtosis is 0.89 < 7, which meets the requirements of sample characteristics.

#### Reliability test

The results of the reliability test analysis are shown in Table 3. The CITC for all variables meets the requirement of greater than 0.5, which indicates that the individual question items are well set. In addition, the Cronbach’s alpha coefficients for each variable meet the basic criterion of greater than 0.7.

#### Validity test

Table 5 shows that the overall KMO value of the sample data is 0.868 with a significance level p-value of 0.000, which is good for validity. The KMO value for each variable is greater than 0.6 with a p-value of 0.000 and the sample data passes the reliability validity test.

**Table 5:** KMO measure and Bartlett’s test.

Indicator	KMO test	Bartlett sphere test		
		Approximate chi-square value	df	Sig.(*p* value)
Psychology	0.877	241.535	190	0
Safety awareness	0.891	293.961	190	0
Evacuation behavior	0.892	259.106	190	0
Social role	0.755	487.665	190	0
Total	0.794	2048.612	190	0

#### Correlation test

In order to further investigate the correlation between the possible behaviors in evacuation and other factors, physical health status, number of training sessions attended, psychological factors, safety awareness, and social roles in the questionnaire were extracted as variables for Spearman correlation analysis, and the results are shown in Table 6.

**Table 6:** Correlation analysis results.

Indicator	Item	Psychology	Safety awareness	Evacuation behavior	Social role
Psychology	Pearson Correlation Significance (two-tailed)	1			
Safety awareness	Pearson Correlation Significance (two-tailed)	0.588** 0.000	1		
Evacuation behavior	Pearson Correlation Significance (two-tailed)	0.431** 0.000	0.618** 0.000	1	
Social role	Pearson Correlation Significance (two-tailed)	0.481** 0.000	0.537** 0.000	0.446** 0.000	1
	N	85	85	85	85

** Correlations are significant at the 0.01 level (two-tailed).

The results of the questionnaire showed that psychological factors, safety awareness, social roles, and related behaviors during evacuation were positively correlated with each other at the 90%-95% confidence interval. The highest correlation coefficient (0.618) was found between safety awareness and evacuation behavior, and the lowest correlation coefficient (0.431) was found between psychological factors and evacuation behavior, which showed that these factors not only influenced each other, but also had an impact on the overall evacuation process.

### Calculation of initial speed based on fuzzy algorithm for factors affecting evacuation behavior

The existing research is mainly based on case collection and experience summary, using qualitative description and analysis methods^60–62^. Although this approach has to some extent revealed the connections among different individual factors of different people, due to the limitations of accuracy and practicality, when dealing with complex data, these tools or methods often fail to achieve precise classification and in-depth analysis. The complex data refers to data related to evacuation behaviors that are multi-source, heterogeneous, high-dimensional, nonlinear, ambiguous, and with uneven sample distribution. Traditional methods that rely on case induction and qualitative description find it difficult to achieve precise classification (such as accurately determining whether an individual belongs to the “rapid evacuation group” or the “slow evacuation group”) and in-depth analysis (such as quantifying the interaction of various factors). Therefore, fuzzy algorithms need to be introduced to handle this uncertainty and complexity, so as to more reasonably calculate the initial evacuation speed. The introduction of fuzzy logic, by establishing the concepts of domain, fuzzy set and affiliation function, effectively transforms individual influencing factors into numerical forms, while integrating them with qualitative analyses of psychological behavior and other variables. By formulating fuzzy rules to predict behavioral outputs, this approach provides both qualitative insights and quantitative results. Therefore, in applications such as traffic flow and pedestrian dynamics within sociophysical models, fuzzy logic facilitates deeper exploration of the mathematical relationship between individual factors and evacuation speed, enabling the transformation of abstract personal attributes into observable and measurable real-world expressions.

In order to quantify the specific effects of different factors on the initial evacuation speed, it is necessary to model the association between individual personnel factors and evacuation speed, including four key factors: psychological factors, safety awareness, evacuation behavior, and social roles^66^, which were obtained as raw data through a questionnaire. A fifth-order Sugeno-type fuzzy system can effectively handle these inputs and yield accurate evacuation speed outputs. A fuzzy algorithm is designed with five input variables and one output variable to achieve a more precise and efficient decision-making process. For the kth fuzzy rule, the construction method is as follows.

The value of the input quantity is* x*_i_=(*i*=1,...,5), and the fuzzy set of each input quantity is denoted as $A_{j}^{{\left( k \right)}} \left( {j = 1, \cdots ,5} \right)$ based on the objective law of the respective set. According to the determination of the influence of each input quantity on the output quantity, the function of the output quantity is obtained, which is denoted by $f_{k} \left( x \right),k = 1,...,M$ In summary, the output of 5th order Sugeno fuzzy logic can be derived as shown in Eqs. (1) and (2) as follows.

1 $$f\left( x \right) = \frac{{\sum\nolimits_{{k = 1}}^{M} {f\left( x \right)\mu _{{A\left( k \right)}} \left( x \right)} }}{{\sum\nolimits_{{k = 1}}^{M} {\mu _{{A\left( k \right)}} \left( x \right)} }}$$

2 $$\mu _{{A^{{\left( k \right)}} }} \left( x \right) = t\left( {\mu _{{A_{1}^{{\left( k \right)}} }} \left( {x_{1} } \right),\mu _{{A_{2}^{{\left( k \right)}} }} \left( {x_{2} } \right), \cdots ,\mu _{{A_{5}^{{\left( k \right)}} }} \left( {x_{5} } \right)} \right)$$

where $t\left( \cdot \right)$ refers to the number of paradigms of t, and $\mu _{{A_{j}^{{\left( k \right)}} }} \left( {x_{j} } \right)$ is the affiliation function of $A_{j}^{{\left( k \right)}}$.

In order to obtain the desired output quantities, four parameters are required, which are the thesis domain of each input quantity, defining a suitable fuzzy set, establishing an accurate affiliation function, and setting appropriate fuzzy rules, which work together to ensure the accuracy and effectiveness of the fuzzy logic system, so that it can more accurately simulate and analyze the uncertainty and fuzziness in real problems^69^.

For every value y in the input quantity theory, there exists a value B(y) between 0 and 1 that corresponds to it. As the value of y changes, these values of B(y) will form a curvilinear function, which is said to be the affiliation function of the value on its set of modes. The affiliation function is used to describe the degree of affiliation between the value y and the set. When B(y) takes the value 1, it means that the value y belongs to the set completely, while when B(y) takes the value 0, it means that the value y does not belong to the set at all. The core principle of fuzzy logic lies in reasoned judgment of uncertain events based on objective laws. This judgment presents the result in the form of a fuzzy set, which transforms this fuzzy result into a specific value. Based on the unique defuzzification system of the Sugeno model, this fuzzy result can be transformed into specific numerical values. Given that the data distribution in this study is uniform, we can use the mean to represent the expected value of the membership function, and the range of the universe of discourse corresponding to the fuzzy set determines the opening size of the membership function.

The core difference between the Sugeno type fuzzy model and traditional fuzzy models like Mamdani lies in the output form and reasoning logic: The Mamdani model outputs as fuzzy sets, which need to go through a defuzzification step to be converted into precise values, and is more suitable for qualitative reasoning scenarios without clear statistical data and relying on empirical rules; while the Sugeno model outputs as linear / constant functions of the input variables, which can directly generate precise values, and has stronger adaptability for coupled calculations with multiple inputs and a single output, can precisely reflect the differential influence weights of various factors through linear weighting, has higher calculation efficiency, and the results are easier to be integrated with engineering models.

This study has chosen the Sugeno model, which has significant advantages in adaptation. Its characteristic of directly outputting precise values enables the conversion of multi-dimensional subjective or objective indicators of personnel directly into specific numerical values that can be embedded in evacuation simulation, without the need for additional processing. This perfectly meets the demand of the simulation for direct input of quantitative parameters. At the same time, this model can accurately fit the algorithm parameters set based on questionnaire statistics, integrating the actual patterns of the survey data into the reasoning process, and is compatible with the variable structure of 5 inputs and 1 output in this study. It can efficiently and accurately perform multi-factor coupling calculations. Its simple numerical reasoning and linearly adjustable characteristics also allow the algorithm results to seamlessly connect with the engineering simulation system of BIM + Pyrosim + Pathfinder, balancing computational efficiency, result accuracy, and model iteration optimization requirements. It is highly consistent with the overall research goal of this study: Quantification of questionnaire data, multi-factor calculation, output precise speed and embedding in engineering simulation.

In this study, the domain range of each input variable refers to the numerical value range set before applying the fuzzy algorithm to quantify the core indicators that affect the evacuation behavior of personnel, such as physical health status and panic level. This range serves as the basic boundary for converting the subjective behavior and objective attributes of personnel from qualitative description to quantitative numerical values that can be calculated by the fuzzy algorithm. The sub-interval division within the domain is the boundary for the fuzzy classification of each indicator. The determination of all domain data and sub-intervals is based on the actual data from the questionnaire survey, combined with the conventional quantitative standards in the research field, the actual laws of personnel evacuation behavior, and the modeling requirements of the fuzzy algorithm.

According to the statistical data, since people tend to experience a high degree of panic during a fire, the initial value of the panic level can be set at 0.5. As the panic situation continues to escalate, the corresponding value will gradually increase until it reaches the maximum value of 1, indicating a state of extreme panic. Physical health conditions have a significant impact on the efficiency of evacuation. Fully capable individuals can assist in escaping, while children and others need to be led to escape, and some individuals who are unable to take care of themselves completely cannot escape independently. Therefore, the threshold is set within the range of 0 to 1. Since each evacuee necessarily has a certain social role, the threshold is set within the range of 0.5 to 1, and different genders have also been differentiated in terms of evacuation ability. The number of times participating in evacuation training is crucial for quickly finding the evacuation route and improving the evacuation efficiency. However, through the questionnaire survey, there are individuals who have never received training at all, while those who have undergone multiple trainings can evacuate more quickly during the escape. Therefore, the threshold is set within the range of 0.5 to 1.

The range of the domain corresponding to the fuzzy set determines the size of the opening of the affiliation function, which is about the expectation of the affiliation function and the size of the opening of the specific values shown in Table 7.

**Table 7:** Expectation and opening size of the affiliation function.

Behavior	Domain	Fuzzy set	Expectation of affiliation function	Opening size
Physical condition	(0,1)	Unable to evacuate independently^*^(0,0.2) Can evacuate with guidance^**^(0.2,0.5) Capable of autonomous evacuation(0.5,1)	0.1,0.35,0.75	(0,0.2) (0.2,0.5) (0.5,1)
Panic level	(0.5,1)	Mild panic(0.5–0.6) Moderate panic(0.6–0.8) Severe panic(0.8-1.0)	0.55,0.7,0.9	(0.5–0.6) (0.6–0.8) (0.8-1.0)
Individual role characteristics^***^	(0.5,1)	Parent (0.5–0.8) Organize (0.5-1.0) Female (0.5-1.0) Male (0.6-1.0)	0.65,0.75,0.75/0.8	(0.5–0.8) (0.5-1.0) (0.5-1.0)/(0.6-1.0)
Evacuation training frequency	(0,1)	Rare (0,0.3) ≤ 2 times (0.3,0.7) > 2 times (0.7,1)	0.15,0.5,0.85	(0,0.3) (0.3,0.7) (0.7,1)

^*^Inability to escape on one’s own means: Early Childhood 0–8 years old (including infants, toddlers and preschoolers), mobility-impaired persons with disabilities, etc.

^**^Led escape: School-age children (6–12 years old) with the ability to run and execute clear instructions, and adults who are unable to judge the external situation on their own but are able to receive and execute instructions.

^***^Individual role characteristics are used to represent the sources of behavioral differences among individuals during evacuation processes. “Parent” and “Organizer” primarily reflect responsibility attributes in evacuation, while ‘female’and “male” primarily reflect action capability characteristics based on group average differences. This paper treats them uniformly as individual role characteristic variables influencing initial evacuation speed and applies fuzzy processing to them.

The input quantities affiliation functions can be determined according to Table 7. For the output quantity its domain and fuzzy set interface need to be determined^64^. According to the above set of correlation and fuzzy rule logic, Matlab software is used to carry out calculations. After entering the affiliation function, the domain of the input quantities and fuzzy sets and other related parameters. The fuzzy toolbox is used to carry out measurements, and the Sugeno model is selected. The initial evacuation speeds of different types of people are obtained through the calculations as shown in Table 8.

**Table 8:** Initial evacuation speeds for different types of people.

Physical Condition	Panic Level	Social role	Evacuation training frequency	Initial evacuation speed(m/s)
Unable to evacuate independently	0.9	0.5	0	0.5
Can evacuate with guidance	0.7	0.8	1	0.8
Capable of autonomous evacuation	0.5	1.0	2	1.2

### BIM model construction

**Fig. 1:**
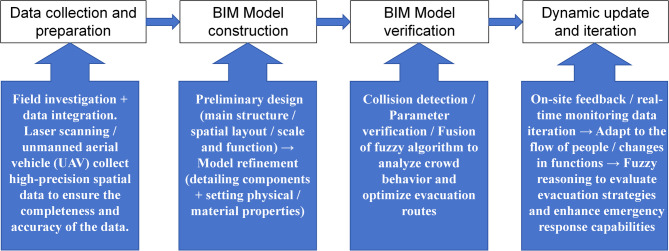
BIM technology application flow chart.

The construction of the BIM model forms the basis for building fire evacuation simulations. It can fully restore the architectural structure, functions, and spatial layout, providing precise data support for optimizing evacuation route planning. The modeling process is divided into five core steps: data collection and preparation, software selection, model construction, model verification, dynamic update and iteration. Each step is well-connected and progressive, ensuring the authenticity and practicality of the model(Fig. 1).

The data collection and preparation stage collects data through on-site investigations combined with building regulations, design drawings, etc., and uses technologies such as laser scanning and drones to collect high-precision spatial data. This ensures the completeness and accuracy of the model data from the source. Software selection uses Autodesk Revit as the core modeling platform. This software supports integrated modeling of architectural structures, mechanical and electrical equipment, and construction processes, and has good compatibility with fuzzy control algorithms, enabling dynamic adjustment and expansion of the model.

Model construction is divided into two stages: preliminary design and refinement. First, the main structure and spatial layout are established, and the overall scale and functional zoning are clarified. Then, elements such as walls, doors and windows, and floors are detailed and their physical and material properties are set, making the model capable of realistic operation. Model verification uses collision detection and parameter verification to avoid design conflicts, integrates resource information through expert reviews, introduces a 4D time dimension to achieve visual simulation of evacuation paths, and integrates fuzzy control algorithms to conduct crowd behavior analysis, optimizing the layout of evacuation channels.

The dynamic update and iteration process continuously optimizes the model based on on-site feedback and real-time monitoring data, accurately reflecting changes in building traffic and functional adjustments. It can also evaluate the effectiveness of various evacuation strategies through fuzzy reasoning, further enhancing the building’s emergency response capabilities and adaptability in emergency situations.

## Experimental design and result analysis

### Case project

**Fig. 2:**
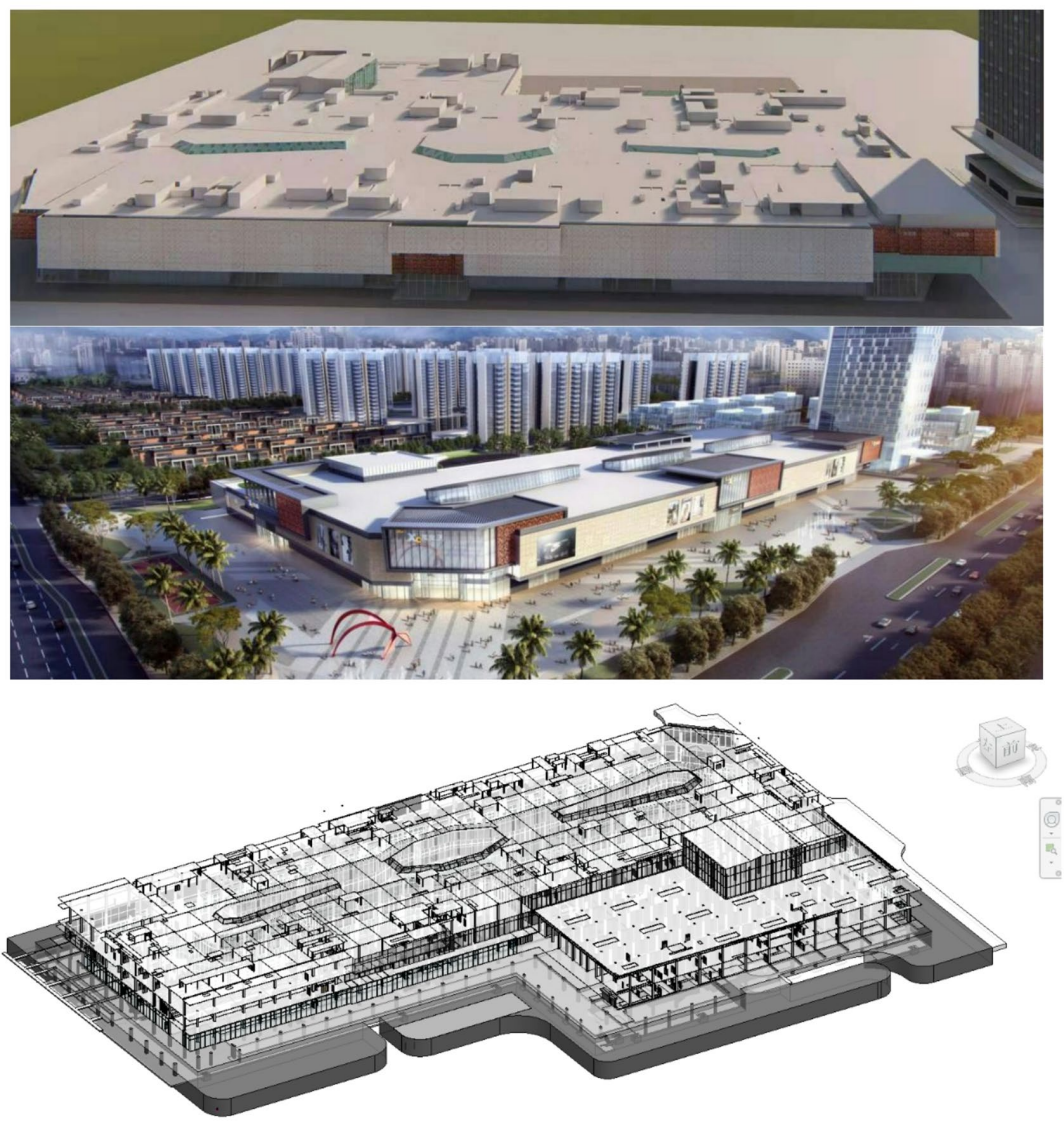
BIM model diagram of the case project.

This paper establishes a fire numerical model based on a large commercial complex building in southwest China. The building area of the project is about 87,000 square meters, with one underground floor and three aboveground floors, with a height of 18 m. All above ground are commercial spaces, including rental stores, amusement parks, theaters, restaurants and other businesses. The BIM building model of the project is shown in Fig. 2.

### Evacuation simulation scheme design

#### Parameter setting

In this paper, fire simulation was performed using Pyrosim software, which is a fire dynamics simulation software based on Fire Dynamics Simulator (FDS) and supports the import of DXF files generated by Revit^66^. In the FDS simulation process, the model mesh is partitioned in order to balance the scientific and computational efficiency. The grid division should be set in combination with the characteristic diameter of the fire source D*, and its specific calculation formula is shown in Eq. (3):

3 $$D^{*} = \left\{ {\frac{Q}{{\rho _{\infty } C_{\infty } T_{\infty } \sqrt g }}} \right\}^{{\frac{2}{5}}}$$

Where *Q* is the heat release rate (kW), with reference to the Technical Standard for Smoke Prevention and Exhaust System in Buildings (GB51251)^67^, taking the value of 3000kW. $\rho$ is the air density, which is about 1.29 (kg/m^3^) at room temperature. *C* is the specific heat of the air, which takes the value of 1.02 kJ/(kg.K) for indoor. *T* is the ambient temperature, which is calculated according to the average value of commercial complexes in Southwest China, taking the value of 298 K (about 25℃). The specific grid parameters are as shown in the Table 9:

**Table 9:** Grid parameter settings.

Grid Name	Grid Boundary Z+/m	Grid Boundary Z-/m	Grid Size/m	Number of Grids
Grid 1	0	3	0.5 × 0.5	21,600
Grid 1	3	6		21,600
Grid 1	6	9		172,800
Grid 1	9	12		21,600
Grid 1	12	15		21,600

#### Scene setting

The fire source area was set to 1 m × 1 m, and the fire growth model was defined as a fast-growing fire, with a fire growth coefficient of 0.0469. Based on the calculation, the time required for the fire source to reach a steady-state heat release rate was 413 s. The initial development stage of the fire was not considered in the simulation, and the total fire simulation time was set to 600 s. To collect data on safety indicators at various interior locations during the fire development process, detectors were placed at fixed positions within the model to record parameters such as temperature, visibility, smoke concentration, and carbon monoxide concentration.

This building is a large shopping mall with a large floor area, and the restaurant on the first floor of the mall is the most likely to have a fire when analyzed in the context of the actual situation. In the event of a fire within the mall, smoke is expected to spread rapidly. Therefore, the fire scenario in this study assumes that the fire originates in the restaurant on the first floor. At the time of ignition, the door to the room is closed, the sprinkler system inside the control room is non-functional, and there is a significant amount of combustible material within the room. The details and core parameters of the fire simulation and evacuation experiment are shown in Table 10:

**Table 10:** Table of operational details and core parameters for fire simulation and evacuation experiment.

Parameter category	Design value	Basis/Verification Method
Fire Source Location	Restaurant Area on the 1st Floor of a Large Commercial Complex in Southwest China	Combined with the characteristics of building formats, the restaurant is a dense area of open flames and flammable materials in commercial buildings, which is a high-fire-risk area; it conforms to the criteria for judging fire hazards in commercial buildings specified in the “Code for Fire Protection Design of Buildings”.
Most Unfavorable Fire Scenario	Closed door at the fire source in the 1st-floor restaurant + failed sprinkler system + more indoor combustibles	An extreme scenario of “uncontrolled fire development + failed fire-fighting facilities + continuous energy release” is selected to maximize the inspection of the safety of evacuation design and the risk resistance of the model, which is consistent with the design principle of the most unfavorable scenario.
Fire Physical Parameter - Heat Release Rate	3000 kW	Refer to the “Technical Standard for Smoke Control and Exhaust Systems in Buildings” (GB51251-2017), matching the heat release characteristics of fires in catering areas.
Fire Physical Parameter - Fire Growth Model	Fast fire model with a growth coefficient of 0.0469	Consistent with the actual working conditions of the restaurant area with many flammable materials and fast fire development speed.
Fire Physical Parameter - Fire Source Area	1 m×1 m	Simulates the actual scale of typical fire points such as kitchen stoves in restaurants.
Fire Physical Parameter - Calculation Time	600s (skipping the initial fire development stage)	Focus on the dynamic changes of the fire scene during the fire development period, taking into account the pertinence of simulation and computational efficiency.
Fire Physical Parameter - Basic Environmental Parameters	Air density: 1.29 kg/m³; Air specific heat: 1.02 kJ/(kg·K); Ambient temperature: 298 K (25℃)	Typical values of the indoor normal temperature environment of commercial complexes in Southwest China.
Evacuation Judgment Parameter - Flue Gas Temperature	≤ 120℃ (critical value)	Above this value, it will repel personnel evacuation and make safe passage impossible, which conforms to the general standard of personnel tolerance limit for fire evacuation.
Evacuation Judgment Parameter - CO Concentration	≤ 0.25% (volume percentage, critical value)	Above this value, personnel will faint due to lack of oxygen and lose the ability to evacuate, which conforms to the general standard of personnel tolerance limit for fire evacuation.
Evacuation Judgment Parameter - Flue Gas Visibility	≥ 10 m (critical value)	Below this value, personnel cannot distinguish directions or see the evacuation path clearly, which conforms to the general standard of personnel tolerance limit for fire evacuation.
Evacuation Judgment Parameter - Safety Judgment Principle	Personnel tolerance limit T = min{T1,T2,T3}; Evacuation is judged safe when ASET> RSET	T1 is the time when the temperature reaches the critical value, T2 is the time when the CO concentration reaches the critical value, and T3 is the time when the visibility reaches the critical value; the classic safety judgment logic for fire evacuation is adopted.
Software Linkage Parameter - Revit and Pyrosim	The BIM model built by Revit is exported as a DXF format file and imported into Pyrosim	Ensure the accurate transmission and consistency of spatial parameters such as building structure, corridors and doors and windows.
Software Linkage Parameter - Pyrosim and Pathfinder	Fire scene environment data (temperature, CO, visibility) from Pyrosim is synchronized to Pathfinder	Realize the linkage simulation of “dynamic changes of fire scene - response of personnel evacuation behavior”, which is consistent with the actual evacuation law.
Rationality Verification of Grid Division	As shown in Table 11	The simulated fire scene parameters diffuse uniformly over time and gradually spread from the fire source floor to the upper floors, which is consistent with the law of large-space fires in commercial buildings; the data of monitoring points changes smoothly without sudden changes, and there is no grid irrelevance error.

#### Conditions for determining evacuation time

According to relevant data, smoke temperature, carbon monoxide (CO) concentration and smoke visibility are the key factors affecting the safety of personnel evacuation^68,69^. Therefore, this paper takes these three indicators reaching the life-threatening threshold as the criteria for determining the safety of personnel’s lives. That is to say, during the evacuation process, as long as the smoke temperature, CO concentration and smoke visibility are still within the safety threshold, personnel can realize safe evacuation:

1. Smoke temperature: when the temperature in the fire is less than 120℃to ensure the smooth passage of personnel, greater than this temperature will be the evacuation of personnel to produce repulsive force^70^.

It should be noted that in the selection of the critical temperature for personnel evacuation, there are two typical thresholds in existing studies:

1. Smoke temperature of 60℃, mainly targeting the hot smoke at the height where people breathe. Exceeding this temperature can easily cause respiratory burns and asphyxiation, which belongs to the inhalation safety critical temperature^71^;
2. Ambient air temperature of 120℃, reflecting the limit that the human body can tolerate for a short period in a hot environment. After exceeding this temperature, the human body will experience significant evacuation repulsion due to intense heat stress and will not be able to pass normally, which belongs to the critical temperature for heat environment tolerance.

The focus of this study is the impact of fire scene environmental temperature on the passage capacity of personnel evacuation routes, and it pays attention to the obstructive effect of the overall heat environment on evacuation, rather than the local risk of inhalation of smoke. Therefore, 120℃ is selected as the critical value for ambient temperature, to determine whether the passage still has the condition for personnel to pass smoothly.

(2) When the volume percentage of CO reaches 0.25%, the personnel will have fainting and other symptoms due to the lack of sufficient oxygen, resulting in the personnel can not be evacuated normally, so the CO concentration should be less than 0.25% in order to ensure that the personnel pass through smoothly.
(3) In the event of a fire after the building structure and area will affect the smoke visibility, after the fire in order to allow personnel to identify the location and see the evacuation path, so the smoke visibility in the mall should be greater than 10 m. Set the time point to reach the critical temperature of 120℃ for the T1, CO concentration to reach 0.25% of the time point for the T2, the smoke visibility is reduced to 10 m of the time point for the T3. in order to ensure that the lives of personnel safety, the personnel tolerance limit should be T = min{T1,T2,T3}.

**Fig. 3:**
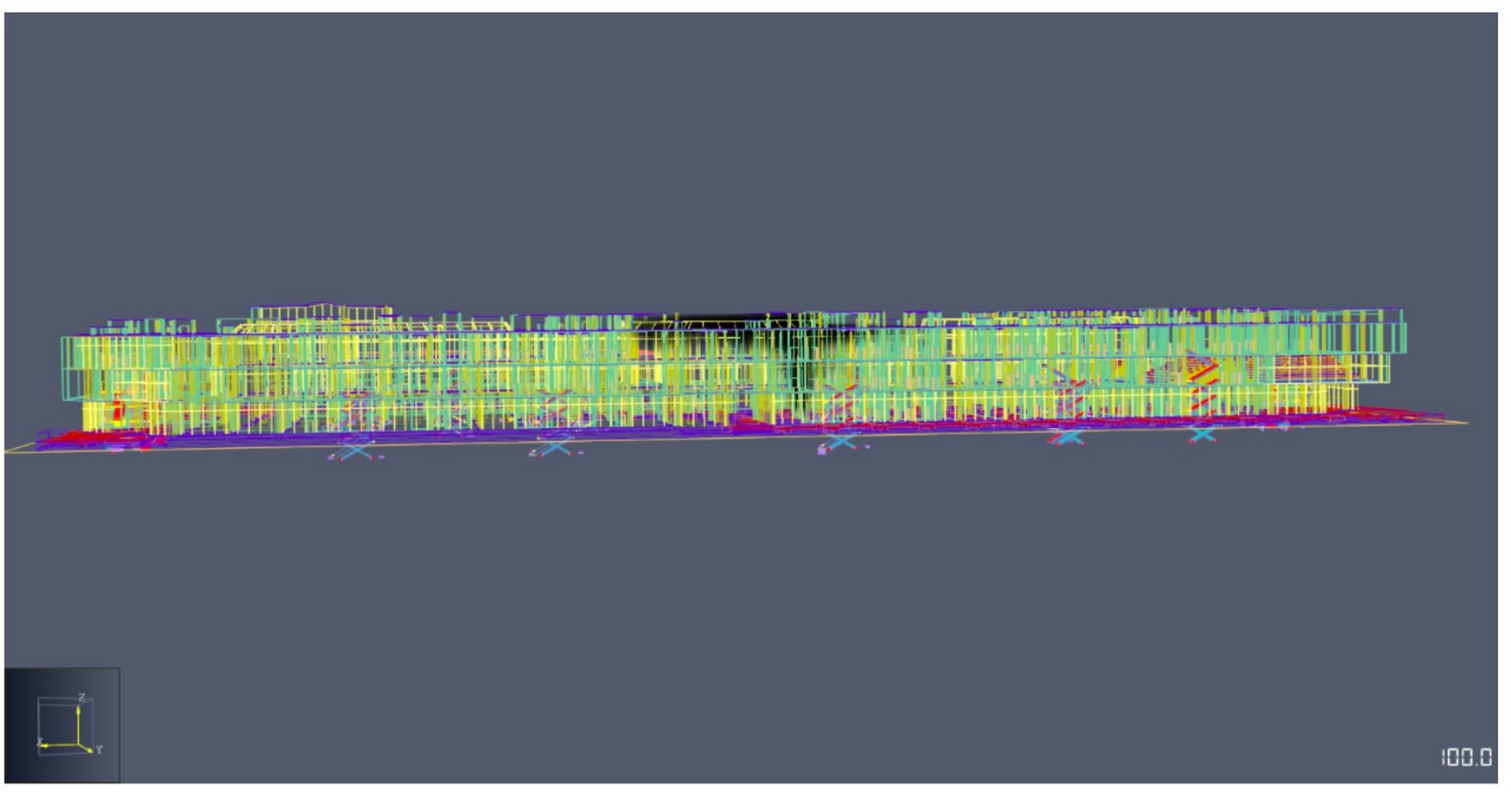
T=100s, fire smoke starts to spread.

**Fig. 4:**
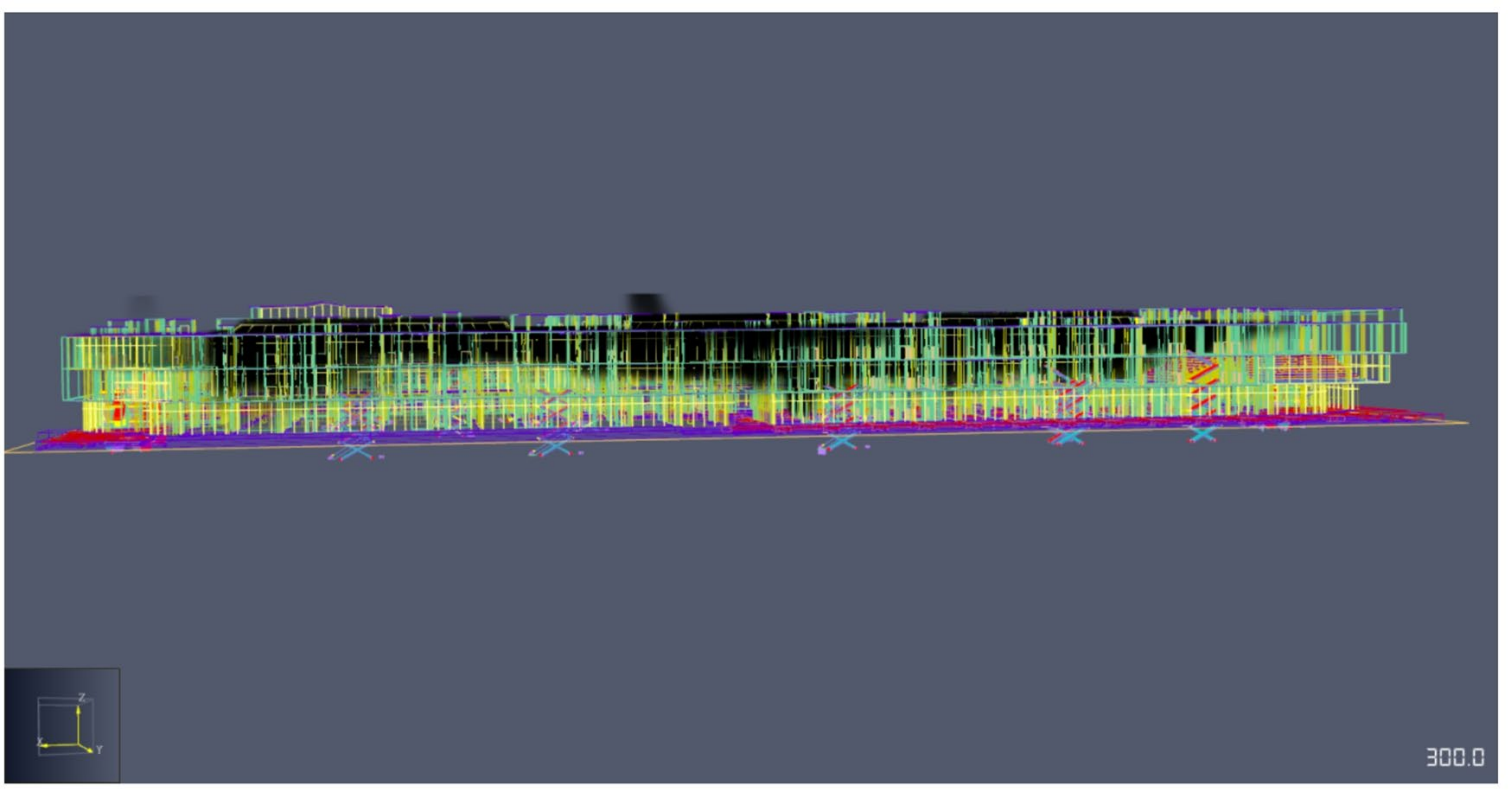
T=300s, fire smoke spreads continuously.

**Fig. 5:**
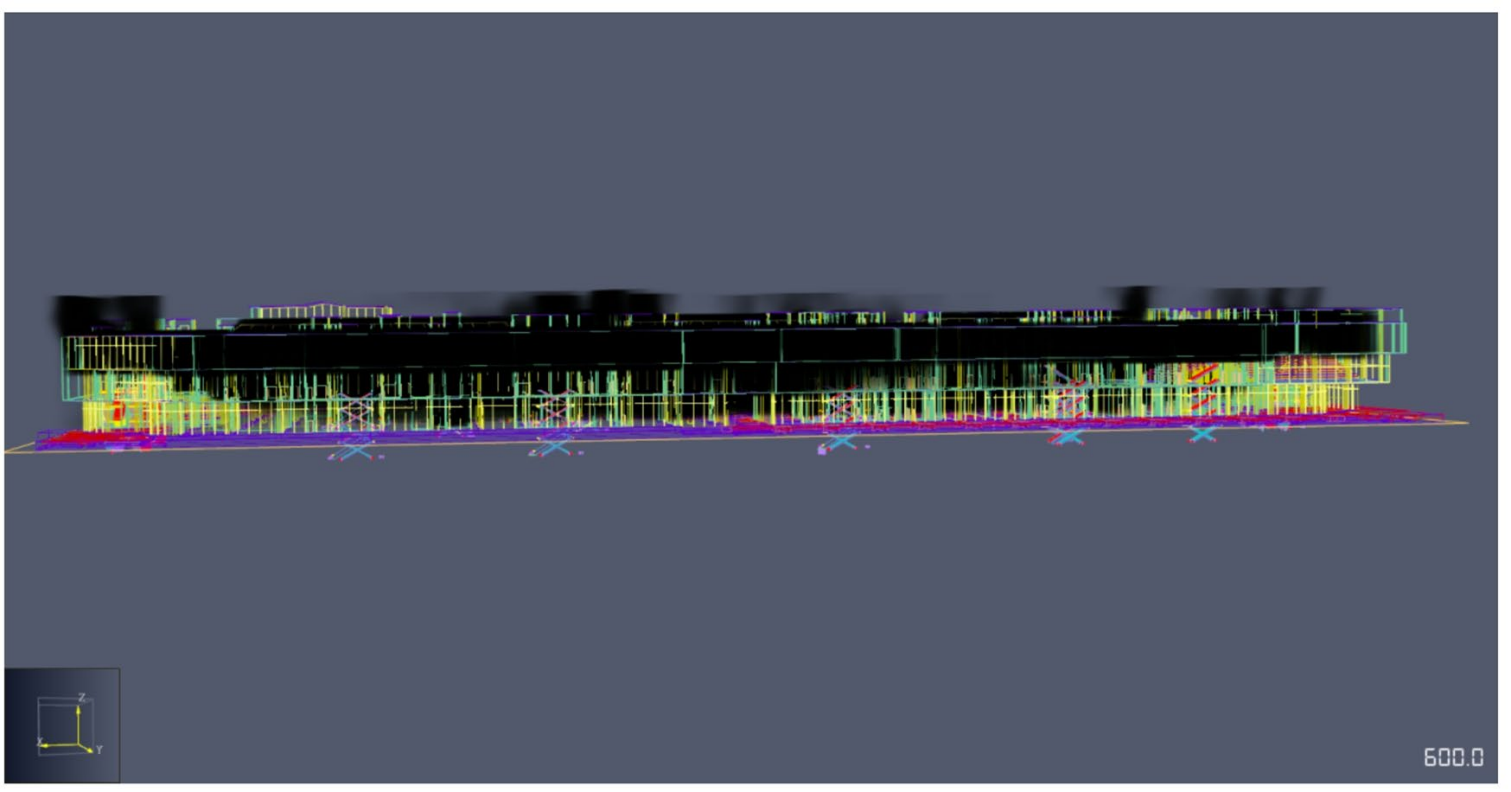
T=600s, fire smoke fills the entire building.

The results of flue gas simulation by Pyrosim software are shown in Fig. 3, 4 and Figure 5.

**Fig. 6:**
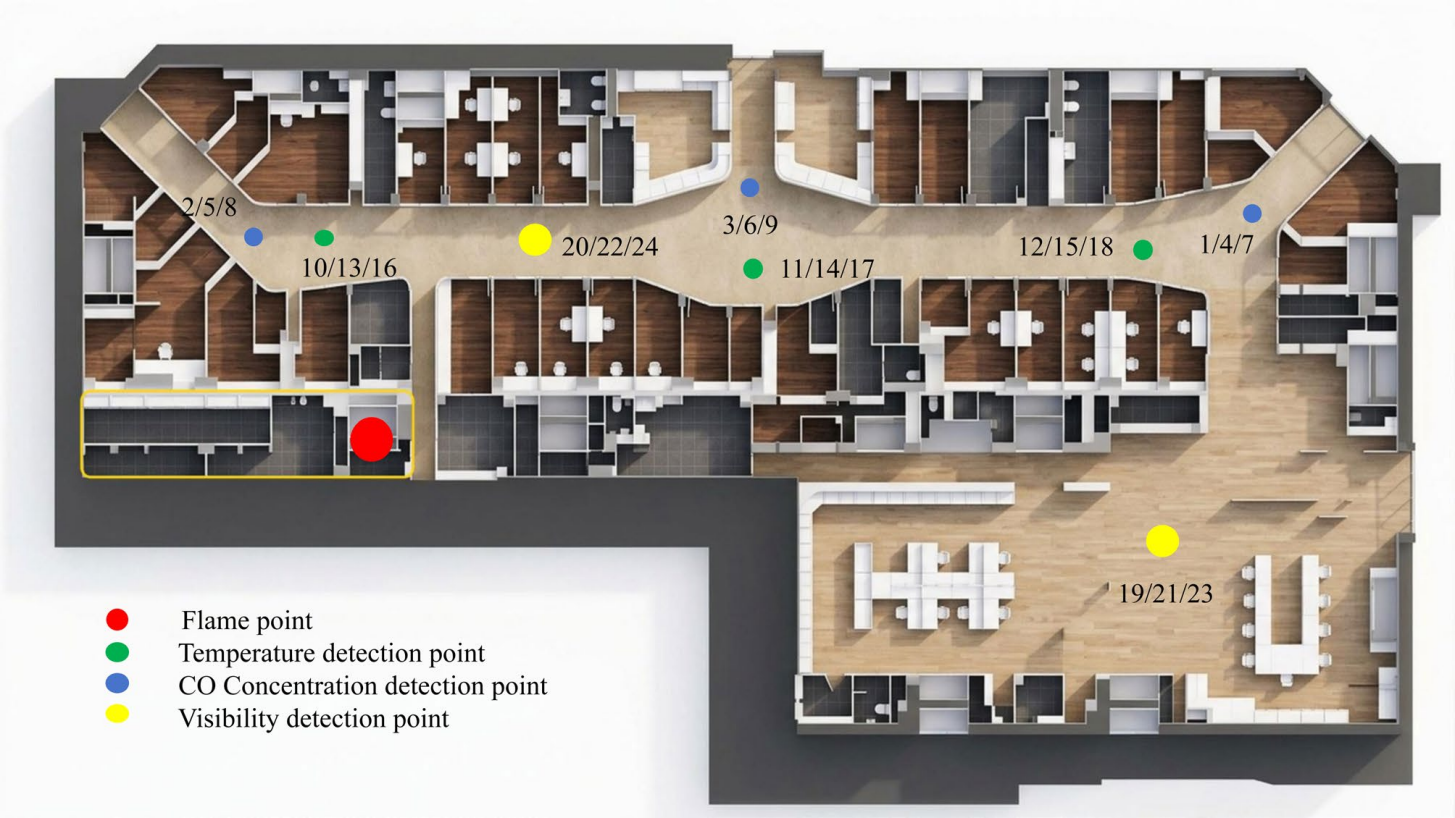
Positioning of detection points.

The monitoring module built into the PyroSim software directly defines the monitoring surfaces/points within the model, without the need for physical equipment. The detection points are set at the breathing height of people on each floor (1.5 m), corresponding to key areas such as the main evacuation passageways and staircases. They are一一matched with the temperature measurement points. The set detection points are shown in Fig. 6.

The simulation software displays the CO concentration values, visibility and temperature values for each floor as shown in Table 11:

**Table 11:** CO Concentration, visibility and temperature values.

Floor	Point	60s	120s	180s	240s	300s	360s	420s	480s	540s	600s
CO(%)											
First floor	1	0.06	0.07	0.08	0.1	0.11	0.13	0.16	0.18	0.21	0.23
	2	0.08	0.1	0.12	0.13	0.15	0.18	0.23	0.25	0.27	0.29
	3	0.07	0.09	0.1	0.11	0.12	0.15	0.17	0.19	0.22	0.25
Second floor	4	0.03	0.04	0.06	0.08	0.1	0.13	0.15	0.17	0.2	0.24
	5	0.06	0.07	0.09	0.11	0.13	0.16	0.19	0.21	0.24	0.26
	6	0.05	0.06	0.08	0.1	0.13	0.15	0.17	0.2	0.22	0.24
Third floor	7	0.02	0.03	0.04	0.06	0.09	0.11	0.13	0.15	0.17	0.19
	8	0.04	0.06	0.08	0.11	0.14	0.17	0.2	0.23	0.27	0.27
	9	0.03	0.04	0.05	0.07	0.08	0.1	0.12	0.14	0.16	0.18
Temperature(℃)											
First floor	10	61	68	75	81	88	97	105	114	121	130
	11	53	57	62	67	76	83	89	96	104	111
	12	46	52	58	66	74	82	91	99	110	120
Second floor	13	40	43	48	52	58	64	70	77	88	100
	14	30	33	36	41	47	54	62	70	79	90
	15	35	38	42	47	54	61	68	75	82	93
Third floor	16	25	26	27	28	29	31	33	35	37	40
	17	24	25	26	27	29	30	31	33	35	37
	18	23	24	26	28	30	33	36	39	43	46
Visibility(m)											
First floor	19	16.2	15.8	15.3	14.5	13.8	13.2	12.4	11.2	10	8.8
	20	15.1	14.5	13.9	13.2	12.3	11.1	10	9.1	8	6.9
Second floor	21	16.8	15.7	15.2	14.7	14.1	13.3	12.5	11.4	10.3	9.0
	22	16.2	15.8	15.4	14.9	14.3	13.6	13	12.1	10.9	9.8
Third floor	23	18.0	17.4	16.9	16.2	15.4	14	13.1	12	10.8	8.9
	24	19.3	19.0	18.6	17.9	17	15.8	14.5	13.1	11.6	9.9

Data analysis indicates that due to the large floor area of the shopping mall and the relatively well-developed ventilation system, heat does not accumulate excessively in any single location. The overall smoke, CO concentration, visibility and temperature all show a trend of diffusion over time. The smoke simulation shows that the overall diffusion rate is more uniform, the first floor, the second floor, the third floor in the cumulative time gradually reached the upper limit of human acceptability. CO concentration begins to approach the critical threshold of 25% starting at 480 s. Temperature at some nodes on the first floor reaches the upper limit of 120 °C around 600 s. Visibility decreases progressively below the acceptable visual threshold of 10 m by 600 s, which aligns with the smoke diffusion simulation results.

The simulation results indicate that the indoor smoke temperature gradually increases over time, with the earliest occurrence of 120 °C recorded at 536 s. The earliest time for the CO concentration to reach 0.25% is 476 s. The earliest time to reach 0.25% is 476 s. The visibility of the flue gas decreases to below 10 m is 418 s, and the record of T3 = 413 s. Therefore, the human tolerance threshold is determined by the minimum of these three values: T = min{T₁, T₂, T₃} = min{536 s, 476 s, 418 s} = 418 s.

### Evacuation simulation results analysis

In this paper, Pathfinder is used to construct a fire evacuation simulation model for the shopping mall. BIM technology was employed to accurately reproduce the architectural information and establish the evacuation scenario, ensuring that the evacuation simulation could be effectively conducted within the mall environment. The BIM model of the shopping mall constructed by Revit is imported into Pathfinder software to complete the construction of the evacuation scene. The initial evacuation speed setting is calculated based on the questionnaire survey data mentioned in point 3 above and through the fuzzy algorithm (as described in Sect.  3.2 of this article), and is set according to the speed values shown in Table 9.

This study classified the evacuated crowd into multiple types based on their physical health conditions, panic levels, social roles, and fire-fighting training experiences. A coupled simulation was conducted using BIM + Pyrosim + Pathfinder + fuzzy algorithm, considering both horizontal and vertical evacuation methods, and the criterion for success was that all personnel were evacuated from the designated area. According to the questionnaire statistics, 82.35% of the simulated population were healthy adults, 15.29% needed assistance, 2.35% were those with mobility impairments, and the gender ratio was 55.29% male and 44.71% female. Through the fuzzy algorithm, three initial evacuation speeds were obtained: 0.5 m/s (unable to escape independently + high panic + no training), 0.8 m/s (able to escape with guidance + moderate panic + a little training), and 1.2 m/s (able to escape independently + low panic + multiple trainings). The safety threshold was set at a temperature of 120℃, a CO concentration of 0.25%, and an visibility of 10 m.

**Fig. 7:**
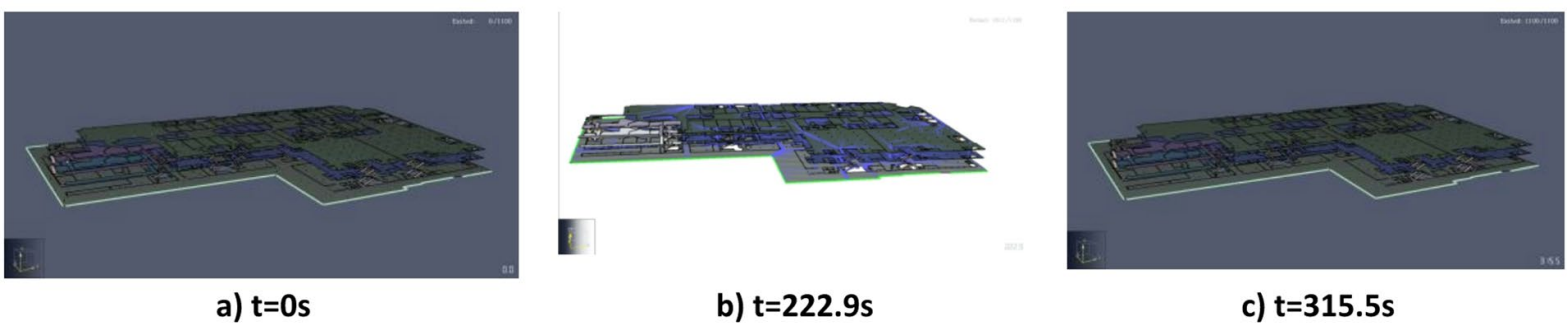
Evacuation simulation effect.

The safety determination for building fire evacuation simulation is based on the numerical comparison between the Available Safe Evacuation Time (ASET) and the Necessary Safe Evacuation Time (NSET) required for evacuation. Specifically, if the ASET exceeds the RSET, it is determined that occupants are able to evacuate safely. Conversely, if ASET is less than RSET, it indicates that some occupants are trapped within the building and unable to reach a safe area, suggesting that the evacuation process requires improvement. Figure 7 shows the simulation of evacuation at different times.

**Fig. 8:**
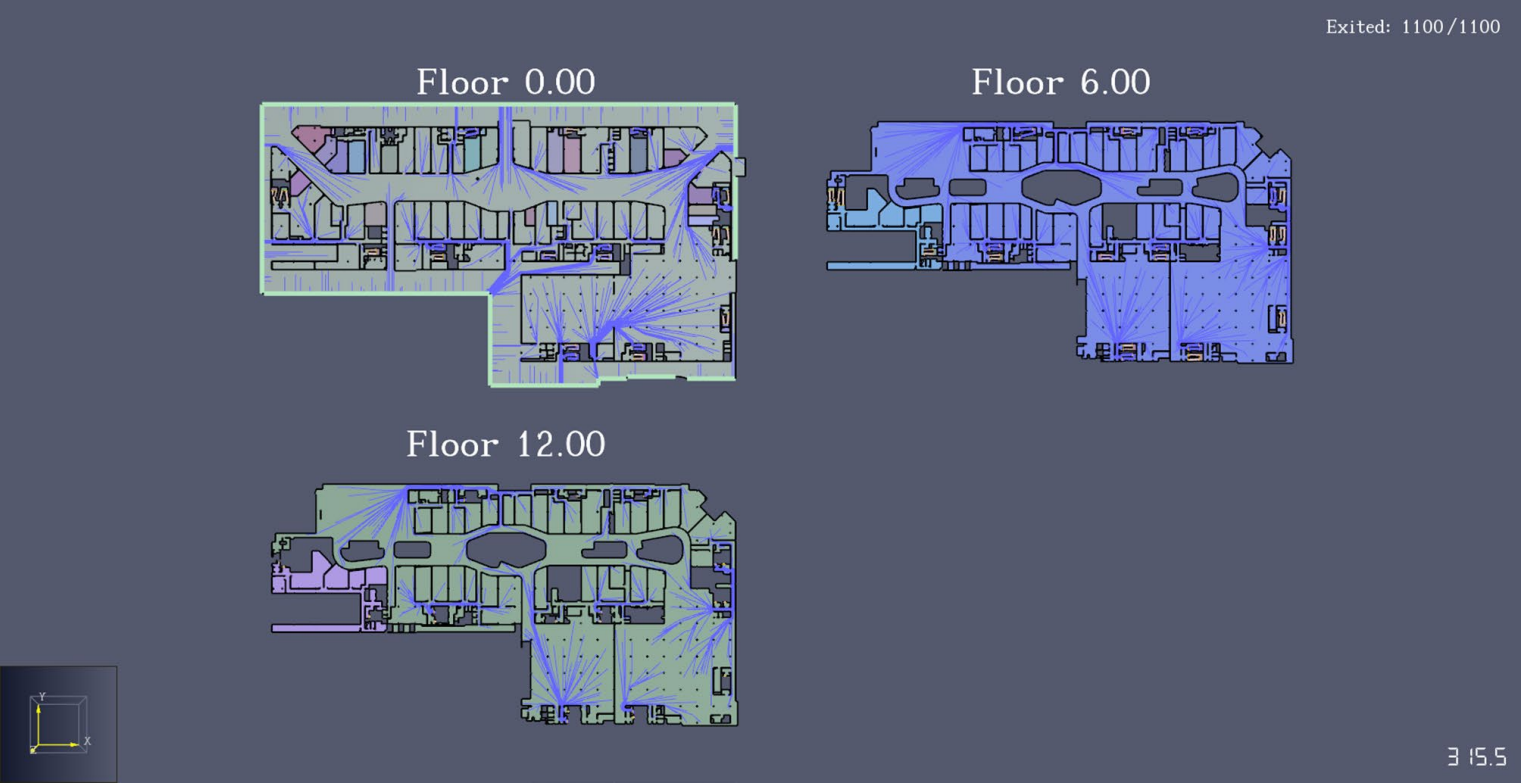
Evacuation routes.

The evacuation path trajectory is shown in Fig. 8.

**Fig. 9:**
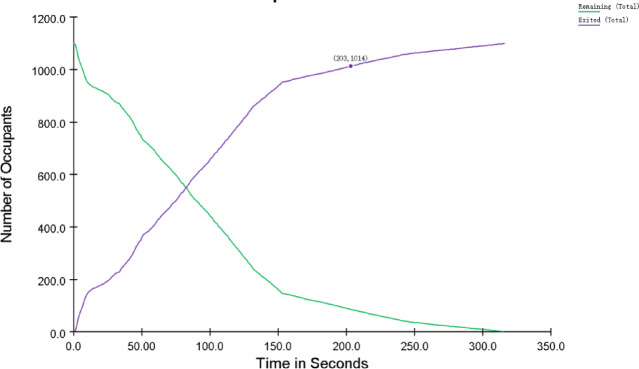
Evacuation result curve.

The dynamics of evacuees over time by analyzing the evacuation data is shown in Fig. 9.

**Fig. 10:**
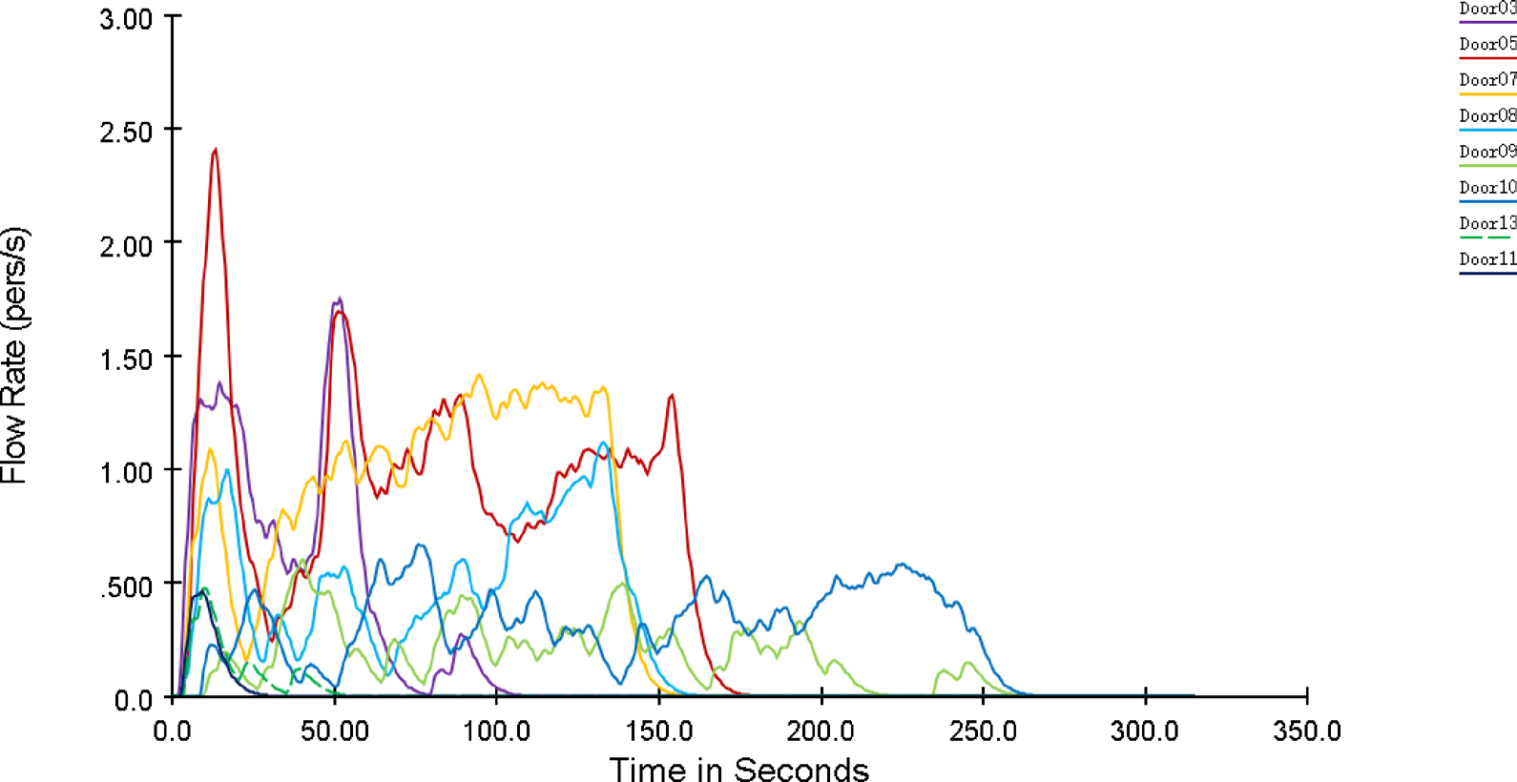
Safety exit clearance rate.

As can be seen from Fig. 8, the evacuation process is a gentle evacuation process and there is no personnel evacuation break. The passage of safety exits is shown in Fig. 10.

As can be seen from Fig. 10, the peak evacuation passage rate among all safe evacuation routes is at Door 5, which occurs at 22.42s, when the passage rate is 2.43 people/s.

Based on the above evacuation simulation analysis, the ASET is determined to be 418 s, while the RSET is 315.5 s. Since the ASET exceeds the RSET, it can be concluded that the occupants are able to evacuate safely.

In summary, the quantitative analysis of the fire status in each area at different time points is shown in Table 12:

**Table 12:** Quantitative Analysis of Fire Status in Each Area at Different Time Points.

Indicator Category	Floor	Data at Different Time Points	Remarks
Carbon Monoxide (CO) Concentration (Volume Percentage)	1st Floor (Fire Source Floor)	60s: 0.06 ~ 0.08%; 476s: 0.25% (critical value); 600s: 0.23 ~ 0.29%	Reaches critical value at 476s
	2nd Floor	60s: 0.03 ~ 0.06%; 600s: 0.20 ~ 0.26%	Below the critical value at 600s
	3rd Floor	60s: 0.02 ~ 0.04%; 600s: 0.17 ~ 0.27%	Local points close to the critical value at 600s
Fire Scene Temperature (℃)	1st Floor (Fire Source Floor)	60s: 46 ~ 61℃; 536s: 120℃ (critical value); 600s: 110 ~ 130℃	Reaches critical value at 536s
	2nd Floor	60s: 30 ~ 40℃; 600s: 82 ~ 100℃	Far below the critical value at 600s
	3rd Floor	60s: 23 ~ 25℃; 600s: 37 ~ 46℃	Minimal impact of fire temperature
Flue Gas Visibility (m)	1st Floor (Fire Source Floor)	60s: 15.1 ~ 16.2 m; 418s: 10 m (critical value); 600s: 6.9 ~ 8.8 m	Reaches critical value at 418s
	2nd Floor	60s: 16.2 ~ 16.8 m; 600s: 9.0 ~ 9.8 m	Close to the critical value at 600s
	3rd Floor	60s: 18.0 ~ 19.3 m; 600s: 8.9 ~ 9.9 m	Local points below the critical value at 600s
Key Time Points of Personnel Tolerance Limit	T1	(Temperature reaches 120℃): 536s (1st Floor, Fire Source Floor)	Critical time point for temperature
	T2	CO concentration reaches 0.25%): 476s (1st Floor, Fire Source Floor)	Critical time point for CO concentration
	T3	Visibility drops to 10 m: 418s (1st Floor, Fire Source Floor)	Critical time point for visibility
	/	Personnel Tolerance Limit (ASET): 418s	Minimum value of T1, T2 and T3

### Discussion

#### Mechanism of RSET reduction via dynamic assignment

RSET is jointly determined by personnel-initiated evacuation and mobile evacuation phases. The mobile phase is influenced not only by individual desired speed but also constrained by congestion, queuing, and bottleneck passage capacity. The role of fuzzy reasoning is not to directly solve for the shortest path, but to map input information, such as psychological factors, safety awareness, individual role characteristics, training experience, and physical condition. This enables the crowd to exhibit speed heterogeneity more consistent with reality during the initial evacuation phase. When the arrival rate at an exit/passage briefly exceeds its capacity, queues and localized high density form, triggering a significant speed decay-congestion amplification effect that ultimately elevates the overall RSET.

The simulation outputs of this paper corroborate the aforementioned bottleneck mechanism: Door 5 serves as the primary passage bottleneck, with its throughput peak occurring at 22.42 s and reaching a maximum of 2.43 persons per second. In evacuation scenarios dominated by such bottlenecks, the sensitivity of RSET typically stems more from bottleneck waiting time than from free-flow walking time. Therefore, the dynamic assignment model achieves indirect compression of bottleneck waiting time by altering the shape of the arrival rate curve, which is the core reason for the approximately 8.7% reduction in RSET.

#### Differences from traditional fixed-speed models

Fixed-speed models typically assign all evacuees to the same or a few discrete speed categories. While this approach is straightforward to implement, it amplifies the illusion of synchronized group movement in complex public buildings: large numbers of individuals arrive at critical junctions within a narrow time window, causing congestion to become highly concentrated. This leads to uneven utilization of exits and excessive queuing at specific points.

The results show that the dynamic assignment model RSET in this paper is 315.5 s. From the finding of the reduction of approximately 8.7%, we can infer that the fixed-speed model RSET is approximately 345.6 s. Regarding safety thresholds, the dynamic assignment model’s ASET-RSET is approximately 102.5 s, while the fixed-speed model corresponds to a safety threshold of around 72 s. This demonstrates that dynamic assignment not only enhances evacuation efficiency but also expands the safety margin under fire uncertainty.

#### Recommendations for evacuation optimization and emergency management

The evacuation safety of complex public buildings depends not only on the number of exits and passage widths, but also on the selection of behavioral parameters. While the overall case study meets the ASET> RSET criterion, Door 5 emerged as a critical bottleneck node. This highlights the need to prioritize load control and diversion guidance at bottleneck nodes during evacuation design and operational management. On one hand, redistributing exit loads can be achieved through optimized wayfinding signage, broadcast guidance, and on-site organization. On the other hand, enhancing safety awareness and reducing irrational hesitation through training and drills improves efficiency during the initial evacuation phase and mitigates bottleneck impacts. Compared to fixed-speed models, dynamic assignment models better reflect the evolution of localized congestion caused by population differences, making them more suitable as auxiliary tools for evaluating and improving evacuation plans in large public buildings.

## Conclusion

### Study findings

This paper focuses on a large commercial complex located in Southwest China. A building model was constructed based on BIM technology, and fire dynamics simulation and evacuation modeling were conducted using Pyrosim and Pathfinder software. Based on the fuzzy algorithm, the uncertainty modeling of the starting evacuation speed is introduced, and the fixed evacuation speed parameters set in the traditional simulation are dynamically adjusted. An integrated evacuation simulation system was developed, incorporating psychological, behavioral, and built environmental factors. A systematic analysis was conducted on the key factors influencing occupant evacuation behavior. The main conclusions are as follows.

1. Based on actual questionnaire data and literature review, this study employs a fuzzy inference approach to dynamically assign initial evacuation speeds for different population groups, which are subsequently applied in Pathfinder evacuation simulations. The simulation results show that the RSET required for evacuation is 315.5s, the minimum critical time (ASET) of fire influencing factors is 418s, and the evacuation process is smooth without congestion and fault phenomenon. Compared with the traditional simulation that sets a fixed starting speed, the dynamic assignment model RSET is shortened by about 8.7%, which verifies the effectiveness of the fuzzy inference algorithm in improving the fidelity of the evacuation simulation.
2. Through reliability and validity testing and Spearman correlation analysis of valid questionnaire data, significant positive correlations were identified between safety awareness, psychological factors, social roles, and evacuation behavior, with correlation coefficients ranging from 0.431 to 0.618. Among them, safety awareness has the most significant effect on evacuation behavior (*r* = 0.618). It indicates that individuals’ judgment, choice of path, and timing of initiating evacuation actions in the face of fire are significantly influenced by psychological dimensions such as their subjective cognition and social roles.
3. Combining the setting of temperature, CO concentration and visibility, three types of fire key influence parameters, T1 (536s), T2 (476s) and T3 (418s) at different time nodes were collected, and the temporal safety boundary of evacuation was clarified. Combined with the evacuation distribution, speed change and exit passage efficiency of personnel at different nodes (the maximum passage rate of 2.43 people/s appeared in Door 5 at 22.42s), a dynamic coupling relationship model between environmental change and crowd response was established, which provided empirical references for the formulation of fire evacuation plan and optimization of emergency response based on BIM.

### Prospects

This paper introduces subjective behavioral perception and dynamic speed modeling in building fire evacuation simulation, which improves the model’s reality perception ability, but there are still deficiencies. On the one hand, the relatively limited number of questionnaire samples and the regional and population homogeneity of the sample sources may affect the representativeness and generalization ability of the behavioral parameters. On the other hand, the quantification and correlation of complex dynamic mechanisms, such as interactions between people and spreading of emotions, are limited, which makes it difficult to completely restore the high uncertainty scenarios of real evacuation processes.

Based on this, subsequent research can conduct in-depth exploration from multiple dimensions. First, further expand the scope of the survey and the sample size, and obtain more representative behavioral data through stratified sampling surveys across regions and among different groups of people, to improve the behavioral parameter system and enhance the universality of model parameters. Second, optimize the construction logic of the fuzzy reasoning system, combine actual evacuation behavior data and objective quantitative methods, correct the rule settings and parameter assignment standards, reduce subjective judgment deviations, and improve the objectivity and accuracy of the model. Third, deepen the research on complex dynamic mechanisms, introduce multi-agent behavior modeling methods, strengthen the quantitative simulation of key processes such as personnel interaction and emotional spread, and more realistically reproduce the uncertainty in the evacuation scenario. Fourth, focus on conducting in-depth research on multi-factor analysis, by constructing multi-factor statistical models, precisely test the independent influence, combined effect, and mutual regulatory effect of each uncertainty factor on evacuation behavior, clarify the complex interaction paths among factors, and provide more scientific and detailed data support and theoretical basis for clarifying the influence mechanism of various uncertainty factors in the actual evacuation process. Fifth, multiple scenarios can be constructed to further compare the impact characteristics of various factors on the evacuation situation in the future, thereby enhancing the multi-dimensional perspective of the research. Overall, through multi-dimensional research, subsequent studies will continuously improve the applicability, accuracy, and real-world explanatory power of the building fire evacuation simulation model, providing more targeted theoretical references and practical support for building fire evacuation design and emergency management.

## Data Availability

The original contributions presented in the study are included in the article/supplementary material, further inquiries can be directed to the corresponding authors.
